# Supplementary material for: Hypertensive Disorders of Pregnancy and Medication Use in the 2015 Pelotas (Brazil) Birth Cohort Study
Source: Int J Environ Res Public Health. 2020 Nov 18;17(22):8541. doi: 10.3390/ijerph17228541 (PMC7698775; doi:10.3390/ijerph17228541)
Supplement: Supplementary file 1 [file ijerph-17-08541-s001.pdf]

Table 1. Variables used for hypertensive disorders of pregnancy (HDP) definition.

**Questionnaires**	**Block**	**Questions related to hypertension (0=No; 1=Yes; 9=IGN)**
**Prenatal follow-up - Full interview (antenatal)**	PREGESTIONAL MORBITIES	*Before this pregnancy you had or had*: (Q46) High blood pressure or hypertension<br>And now, during this pregnancy, so far, you have presented any of these health problems: (Q61a.) Hypertension
**Perinatal**	PRENATAL BLOCK AND GESTATIONAL MORBIDITY	*Now let's talk about some diseases that may have occurred during pregnancy. During the pregnancy....* (Q106). Did you have high blood pressure? IF NOT OR IGN go to 108<br>(Q107). Did you have high blood pressure before pregnancy?<br>(Q108). Did you have eclampsia or pre-eclampsia?

Full questionnaires available in Portuguese. URL: http://epidemio-ufpel.org.br/site/content/coorte_2015-en/questionnaires.php

Supplementary material 2

HDP classification:

- Chronic hypertension was attributed to those who answered yes to questions about hypertension before the current pregnancy in either the antenatal or perinatal components of the questionnaires.
- Those who had chronic hypertension and answered yes to the question of preeclampsia in the perinatal component were classified as having preeclampsia superimposed on chronic hypertension.
- Women who answered yes to hypertension in the current pregnancy, without previous hypertension or preeclampsia were classified as having gestational hypertension.
- All those women who answered yes for preeclampsia, and no history of gestational hypertension, chronic hypertension or preeclampsia superimposed on chronic hypertension were classified as having preeclampsia.

Table S3: Guidance for the drug treatment of hypertension in pregnancy

**Guidance for the drug treatment of hypertension in pregnancy**	**Febrasgo 2011[1]**	**High-risk Gestional Manual[2]**
Definition of hypertension	Hypertension, when systolic blood pressure reaches ≥ 140 mmHg and/or diastolic blood pressure, reaches ≥ 90 mmHg, in two measurements with an interval of at least four hours.	Blood pressure equal to or greater than 140/90mmHg based on the mean of at least two measurements.
Initiation of antihypertensive treatment	In chronic hypertensive pregnant women, who start prenatal care before the 20th week, we use hypotensive drugs whenever diastolic blood pressure is equal to or greater than 90 mmHg. The therapeutic goal will, therefore, be the normalization of blood pressure.	The pressure values (in mmHg) for maintenance or reinstitution of drug therapy are 150 to 160 for systolic or 100 to 110 for diastolic or the presence of a lesion in target organs such as left ventricular hypertrophy or renal failure.
Selection of the drug	Several drugs can be used by the clinician, among which we can mention methyldopa, pindolol (beta-blocker with intrinsic sympathomimetic activity), hydralazine, prazosin, calcium channel antagonists. In cases where the patient, chronic hypertensive, starts the pregnancy under the use of antihypertensive therapy and is adequately treated and controlled the medication should be maintained even if such a drug is a diuretic. Drugs: Beta-blocker - Pindolol: 10 to 30 mg/day or Methyldopa: 0.5 to 2 g/day with addition of Amlodipine: 5 to 20 mg/day	Methyldopa is the drug of choice as first-line therapy. In the case of refractoriness to therapy, consider the possibility of salt retention and in this case, a diuretic can be added to the regimen. Other agents that can be used are beta and alpha-adrenergic blockers. Experience with calcium antagonists has been growing lately, and no significant adverse effects have been reported with their use. The use of diuretics, when indicated, is safe and effective, may potentiate the response of other antihypertensive agents and is not contraindicated, except in situations where the uterus-placental perfusion is already reduced (preeclampsia and fetal growth restriction). Diuretics should not be started as an outpatient treatment drug during pregnancy but can be maintained if the woman is pregnant and well-controlled.
Contraindications	Due to their harmful effects on the fetus, angiotensin conversion enzyme (ACE) inhibitors such as captopril and angiotensin II antagonists, such as losartan, should be replaced. Also, the association of propranolol and diuretic can be replaced by pindolol or methyldopa with advantages for fetal interests.	Pregnant women who are well controlled with antihypertensive medication before pregnancy may be maintained with the same agents, except angiotensin-converting enzyme inhibitors and A-II receptor antagonists.

References:

[1] Federação Brasileira das Associações de Ginecologia e Obstetrícia (FEBRASGO), Manual de Gestação de Alto Risco, 2011.

[2] Ministério da Saúde, Gestação de alto risco: manual técnico, 5 ed., Ministério da Saúde, Secretaria de Atenção à Saúde, Departamento de Ações Programáticas Estratégicas., Brasília, 2012.

Table 4. Characteristics of women excluded due to incomplete data on HDP (n = 8).

ID*	Hypertension -antenatal component	Hypertension - perinatal component	Preeclampsia	Antihypertensive use
307	No	No	ignored	No antihypertensive use
345	No	missing	missing	Ignored, not known
822	missing	No	ignored	Any other antihypertensive without methyldopa
918	missing	no	ignored	No antihypertensive use
1083	missing	no	ignored	Ignored, not known
1577	missing	yes	no	Methyldopa monotherapy - first to the third trimester
2201	missing	ignored	no	Ignored, not known
4121	missing	ignored	ignored	No antihypertensive use

* IDs are de-identified.

Table 5. Characteristics of antihypertensive therapy during pregnancy in women in the Pelotas cohort 2015, according to the type of hypertensive disorder of pregnancy (HDP), including missing and those which HDP was unknown. (N=4270)*.

**Characteristic**	**Total**	**No antihypertensive drug**	**Methyldopa monotherapy**	**Any other antihypertensive drug without methyldopa**	**Any other antihypertensive drug+methyldopa**	**Missings or ignored**
	(N = 4270)	(N=3377)	(N=219)	(N=358)	(N=84)	(N=232)
**No HDP, n (%)**	2926 (68.5)	2482 (84.8)	1 (0.0)	264 (9.0)	1 (0.0)	178 (6.1)
**Type of HDP**, n (%)**						
Chronic hypertension	409 (9.6)	235 (57.5)	83 (21.3)	30 (7.3)	45 (11.0)	16 (3.9)
Gestational hypertension	636 (14.9)	493 (77.5)	60 (9.4)	45 (7.1)	15 (2.4)	23 (3.6)
Preeclampsia superimposed on chronic hypertension	89 (2.1)	29 (32.6)	34 (38.2)	7 (7.9)	14 (15.7)	5 (5.6)
Preeclampsia-eclampsia	191 (4.5)	132 (69.1)	35 (18.3)	11 (5.8)	8 (4.2)	5 (2.6)
Hypertension during pregnancy with preeclampsia unknown	11 (0.3)	3 (27.3)	5 (45.4)	0	1 (9.1)	2 (18.2)
**HDP unknown, n (%)**	8 (0.2)	3 (37.5)	1 (12.5)	1 (12.5)	0	3 (37.5)

* Includes all women eligible. **Adapted according to recommended by the FEBRASGO and Ministry of Health guide; 9 hypertension cases not possible to classify because there was no information about eclampsia/preeclampsia.

Table 6. Antihypertensive medication use by trimester by women **without HDP** in the 2015 Brazil Birth Cohort according to ATC code and substance name. N=2748*.

ATC4 - Definition	Substance name	First trimester		Second trimester		Third trimester	
		n	%	n	%	n	%
B01AC - Platelet aggregation inhibitors excl. Heparin	Acetylsalicylic acid	25	0.9	17	0.6	11	0.4
C02AB - Antiadrenergic agents, centrally acting	Methyldopa	2	0.1	2	0.1	2	0.1
C10AX - Other lipid modifying agents	Omega-3	30	1.1	73	2.7	125	4.5
C04AA - 2-amino-1-phenylethanol derivatives	Isoxsuprine	12	0.4	16	0.6	34	1.2
C08CA - Dihydropyridine derivatives	Nifedipine or amlodipine	0	0	10	0.4	22	0.8
C02XX - ANTIHYPERTENSIVES**		0	0	0	0	1	0.0
C05CA - Bioflavonoids	Diosmin	0	0	4	0.1	4	0.1
C03AA - Thiazides, plain	Hydrochlorothiazide	0	0	0	0	0	0
C09AA - ACE inhibitors, plain	Captopril or enalapril	0	0	0	0	0	0
C09CA - Angiotensin II receptor blockers (ARBs), plain	Candesartana or losartana	0	0	0	0	0	0
C07AB - Beta blocking agents, selective	Atenolol or metoprolol	1	0	0	0	0	0
C03CA - Sulfonamides, plain	Furosemide	1	0	0	0	0	0
C10AA - HMG CoA reductase inhibitors	Synvastatine	0	0	0	0	0	0
C01BD - Antiarrhythmics, class III	Amiodarone	1	0	1	0	1	0
C01CA - Adrenergic and dopaminergic agents	Ethylphrine	1	0	2	0.1	0	0
C02DB - Hydrazinophthalazine derivatives	Hydralazine	0	0	0	0	0	0
C03BA - Sulfonamides, plain	Chlortalidone	0	0	0	0	0	0
C03XX - DIURETICS**		0	0	0	0	0	0
C07AA - Beta blocking agents, non-selective	Pindolol, propranolol or sotalol	1	0	1	0	0	0
C10AB - Fibrates	Ciprofibrate	1	0	0	0	0	0
C01AA - Digitalis glycosides	Digoxin	0	0	0	0	0	0
C01XX - CARDIAC THERAPY**		0	0	0	0	0	0

* 178 missings - medication missing or ignored.

** Substance name not provided.

Table 7. Antihypertensive medication use by trimester by women **with HDP** in the 2015 Brazil Birth Cohort according to ATC code and substance name. N=1285*.

ATC4 - Definition	Substance name	First trimester		Second trimester		Third trimester	
		n	%	n	%	n	%
B01AC - Platelet aggregation inhibitors excl. heparin	Acetylsalicylic acid	31	2.4	45	3.5	31	2.4
C02AB - Antiadrenergic agents, centrally acting	Methyldopa	148	11.5	200	15.6	251	19.5
C10AX - Other lipid modifying agents	Omega-3	12	0.9	23	1.8	26	2.0
C04AA - 2-amino-1-phenylethanol derivatives	Isoxsuprine	7	0.5	8	0.6	15	1.2
C08CA - Dihydropyridine derivatives	Nifedipine or amlodipine	1	0.1	4	0.3	17	1.3
C02XX - ANTIHYPERTENSIVES**		2	0.2	5	0.4	15	1.2
C05CA - Bioflavonoids	Diosmin	2	0.2	1	0.1	3	0.2
C03AA - Thiazides, plain	Hydrochlorothiazide	13	1.0	4	0.3	0	0
C09AA - ACE inhibitors, plain	Captopril or enalapril	13	1.0	7	0.5	3	0.2
C09CA - Angiotensin II receptor blockers (ARBs), plain	Candesartana or losartana	10	0.8	2	0.2	1	0.1
C07AB - Beta blocking agents, selective	Atenolol or metoprolol	9	0.7	1	0.1	0	0
C03CA - Sulfonamides, plain	Furosemide	1	0.1	0	0	0	0
C10AA - HMG CoA reductase inhibitors	Synvastatine	2	0.2	1	0.1	1	0.1
C01BD - Antiarrhythmics, class III	Amiodarone	0	0	0	0	0	0
C01CA - Adrenergic and dopaminergic agents	Ethylphrine	0	0	0	0	0	0
C02DB - Hydrazinophthalazine derivatives	Hydralazine	1	0.1	0	0	2	0.2
C03BA - Sulfonamides, plain	Chlortalidone	1	0.1	0	0	1	0.1
C03XX - DIURETICS**		1	0.1	2	0.2	2	0.2
C07AA - Beta blocking agents, non-selective	Pindolol, propranolol or sotalol	0	0	0	0	1	0.1
C10AB - Fibrates	Ciprofibrate	0	0	0	0	0	0
C01AA - Digitalis glycosides	Digoxin	0	0	1	0.1	0	0
C01XX - CARDIAC THERAPY**		0	0	0	0	1	0.1

* 51 missings - medication missing or ignored. ** Substance name not provided.
